# A Novel Entity of Renal Toxicity After Combination Chemotherapy: An Onco-Nephrology Kidney Biopsy Teaching Case

**DOI:** 10.1016/j.xkme.2026.101439

**Published:** 2026-06-16

**Authors:** Gian Marco Berti, Federica Maritati, Francesco Tondolo, Stefano Chilotti, Benedetta Fabbrizio, Gianandrea Pasquinelli, Gaetano La Manna, Gisella Vischini

**Affiliations:** 1 Department of Medical and Surgical Sciences (DIMEC), Alma Mater Studiorum–University of Bologna, Bologna, Italy; 2 Nephrology, Dialysis and Kidney Transplant Unit, IRCCS Azienda Ospedaliero Universitaria di Bologna, Bologna, Italy; 3 Pathology Unit, IRCCS Azienda Ospedaliero Universitaria di Bologna, Bologna, Italy

**Keywords:** Onco-nephrology, cryoglobulinemic glomerulonephritis, kidney biopsy, chronic thrombotic microangiopathy, myeloid bodies

## Abstract

Renal injury associated with combination cancer therapies represents a growing diagnostic challenge. Kidney biopsy enables early and appropriate detection of these cases, helping in accurate differential diagnosis and individualized management in these increasingly frequent and complex clinical settings. We report a tricky onco-nephrology teaching case involving a 65-year-old woman with recurrent high-grade Müllerian carcinoma who developed progressive kidney dysfunction, arterial hypertension, and new-onset subnephrotic range proteinuria during treatment with carboplatin, gemcitabine, and bevacizumab. Serologic tests were negative for autoimmune, viral, and hematologic causes. Kidney biopsy showed an advanced membranoproliferative pattern with eosinophilic and periodic acid–Schiff positive intracapillary deposits, diffuse endothelial injury, and unusual tubular myelin figures. Two weeks after kidney biopsy, the patient developed cutaneous purpura that shifted the diagnostic framework toward a systemic vasculopathy. This was supported by the ultrastructural evidence of polyclonal IgM-dominant organized intraluminal deposits and consistent with a drug-induced cryoglobulinemic glomerulonephritis superimposed on gemcitabine-induced chronic thrombotic microangiopathy with unexpected renal lipidosis. To our knowledge, this represents the first reported case of this specific renal injury profile in this clinical setting, underscoring the critical role of kidney biopsy in optimizing personalized care within the emerging field of onco-nephrology.

Advances in oncologic therapies have substantially improved survival, increasing the need for close interdisciplinary collaboration in the care of patients with cancer. Kidney involvement is a frequent and clinically relevant complication in this population, resulting from paraneoplastic mechanisms, direct tumor-related effects, or toxicity of anticancer therapies. The rapid expansion of combination and targeted treatments has broadened therapeutic options for solid and hematologic malignancies but has also introduced novel and overlapping patterns of kidney injury, posing new diagnostic and management challenges for nephrologists and nephropathologist.^1^^,^^2^ In this complex clinical context, kidney impairment often presents with nonspecific features, making etiologic distinction challenging. Multiple pathogenic mechanisms, including immune complex-mediated processes, drug-induced toxicity, endothelial injury, and renal cellular inclusions may coexist, resulting in composite and atypical histological patterns that expand the spectrum of recognized renal pathologies associated with anticancer therapies. In such cases, kidney biopsy, particularly when complemented by ultrastructural analysis and careful clinicopathologic correlation, is essential for accurate diagnosis and tailored management.^3^^,^^4^

To our knowledge, we report the first case of a drug-induced cryoglobulinemic glomerulonephritis (CryoGN) associated with gemcitabine-related chronic thrombotic microangiopathy (cTMA) and renal lipidosis in a patient treated with carboplatin, gemcitabine, and bevacizumab, underscoring the importance of timely kidney biopsy in personalized oncological care.

## Case Report

### Clinical History and Initial Laboratory Data

**Figure 1:**
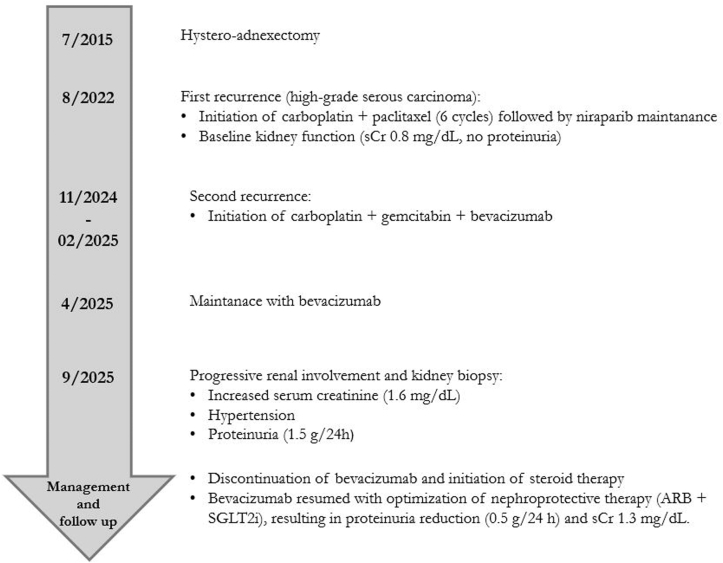
Clinical timeline of oncologic treatment and kidney involvement.

A 65-year-old White woman with recurrent high-grade Müllerian carcinoma was referred to Onco-Nephrology Outpatient Clinic for progressive kidney impairment and new-onset proteinuria. She underwent hystero-adnexectomy in 2015, with recurrence in 2022 (high-grade serous carcinoma with peritoneal carcinomatosis, BRCA wild type) treated with chemotherapy and niraparib maintenance. Further progression occurred during the last year with common iliac lymph node involvement. She received carboplatin, gemcitabine, and bevacizumab, followed by maintenance therapy with bevacizumab alone. Her baseline serum creatinine level was 0.8 mg/dL without proteinuria. From 6 months after gemcitabine initiation, creatinine levels progressively increased to 1.6 mg/dL alongside arterial hypertension and subnephrotic proteinuria (1.5 g/24 h, Fig 1). Serologic testing was negative for viral infections, autoimmune diseases, and hematologic disorders (Table 1, Box 1). Imaging performed for oncologic surveillance and clinical follow-up showed no lymphadenopathy or extrarenal manifestations, further supporting the exclusion of a systemic autoimmune or lymphoproliferative process.

**Table 1:** Patient’s Laboratory Data Before and at Admission.

Test	Time of Test		
	Before admission	At Admission (KB)	Reference Range
Hemoglobin, g/dL	12.9	12	13-17.5
WBC count (cells /μL)	6.26	5,67	3.6-10.5
Platelet count (cells /μL)	280	218	150,000-450,000
Reticulocyte count (%)	N.A.	2.6	0.5-2
sCr, mg/dL	0.8	1.6	0.50-1.00
eGFR^a^, mL/min/ 1,73 m^2^	82	36	>90
Sodium, mEq/L	143	140	135-148
Potassium, mEq/L	4.3	4.7	3.5-5.1
Albumin, g/dl	44	39	3.5-5.2
Biliruubin direct/indirect	0.07/0.34	0.05/0.29	<0.3/0.9
LDH, IU/L	211	264	<248
Haptoglobin, g/L	N.A.	80	30-200
Schistocytes (%)	N.A.	Negative	Negative
Proteinuria, mg/24 h	90	1500	<200
Cryoglobulin	N.A.	Negative	Negative
Hematuria, RBC/hpf	N.A.	9	<15
Rheumatoid factor, U/mL	N.A.	Positive	Negative
C3, mg/dL	N.A.	123	90-140
C4, mg/dL	N.A.	9	10-40
ANA, U^b^	N.A.	Negative	Negative
HCV/HBV/HIV, copies/mL	Negative	Negative	Negative
CMV/EBV/BK, copies/mL	Negative	Negative	Negative
SARS-CoV-2	Negative	Negative	Negative
k/ λ Ratio	1.4	1.6	0.26-1.65
SPEP	Negative	Negative	Negative
UPEP	Negative	Negative	Negative
S/U IFE	Negative	Negative	Negative

Abbreviations: ANA, antinuclear antibodies; CMV, Cytomegalovirus; EBV, Epstein-Barr virus; HBV, hepatitis B virus; HCV, hepatitis C virus; HIV, human immunodeficiency virus; hpf, high power field; KB, kidney biopsy; RBC, red blood cell; SPEP, serum protein electrophoresis; S/U IFE, serum and urine immunofixation electrophoresis; UPEP, urine protein electrophoresis; WBC, white blood cells.

a Calculated according to the 2021 CKD-EPI.

b ANA- associated autoantibodies (including anti-ds-DNA).

Box 1 **Home medications.**

Olmesartan (oral), 40 mg/d

Amlodipine (oral), 10 mg/d

Doxazosin (oral), 4 mg/d

Furosemide (oral), 50 mg/d

Allopurinol (oral), 300 mg/d

Sodium zirconium cyclosilicate (oral), 5 g on alternate d

### Kidney Biopsy

**Figure 2:**
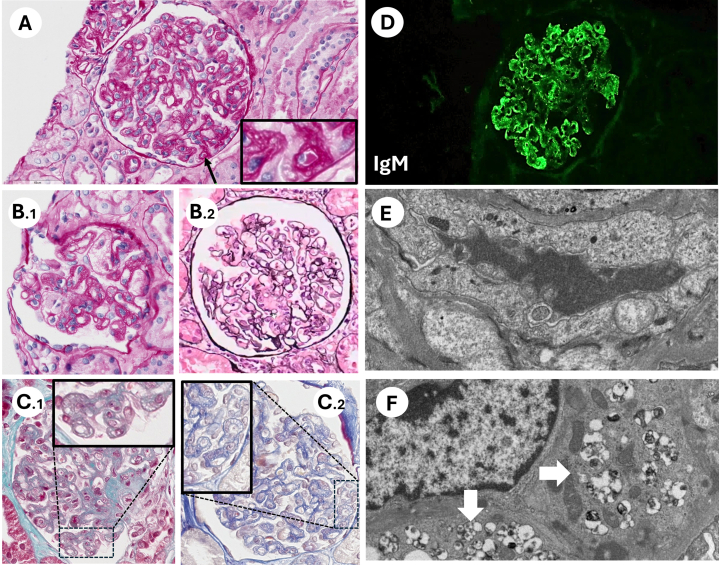
Kidney biopsy. (A) Periodic acid–Schiff (400×), diffuse double contours, mild global mesangial matrix expansion with occasionally intraluminal foam cells and periodic acid–Schiff positive hyalin thrombi (black arrow). (B) 1, Periodic acid–Schiff (400×); 2, Jones methenamine silver (400×); mesangiolysis, and bloodless capillary loop aneurysms. (C) 1, Masson's Trichrome stain (400×); 2, Acid fuchsin orange G-stain (400×); diffuse double contours with occasionally cellular interposition. (D) Immunofluorescent staining, global granular IgM positivity in mesangium and capillary loops, and occasionally IgM positive intraluminal material. (E) Electron Micrographs, intraluminal organized Cryoplug. (F) Electron micrograph (7,900×), myelin bodies (white thick arrows).

Kidney biopsy was performed and light microscopy showed an advanced membranoproliferative (MPGN) pattern with low cellularity and diffuse double-contour formation of the glomerular basement membranes (GBM), associated with intraluminal eosinophilic, periodic acid–Schiff positive hyalin thrombi corresponding to dominant polyclonal IgM deposits on immunofluorescence (Fig 2). Ultrastructural findings of diffuse mesangiolysis with endothelial cell loss supported a chronic microangiopathic process. Acute tubular injury with myelin figures in tubular epithelial cells and moderate arteriosclerosis were also present.

### Diagnosis

**Figure 3:**
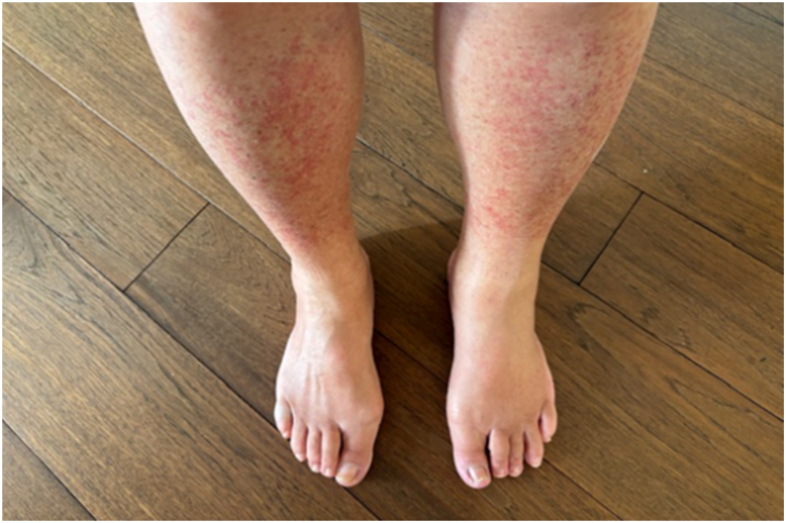
Purpuric bilateral cutaneous lesions.

Two weeks after the preliminary kidney biopsy results, the patient developed diffuse bilateral lower-extremity cutaneous purpura, shifting the diagnostic framework toward a systemic vasculopathy consistent with CryoGN (Fig 3). This was confirmed by ultrastructural evidence of the organized intraluminal deposits, in the presence of low C4 levels and elevated rheumatoid factor, despite serial circulating cryoglobulin assays repeatedly testing negative. Cutaneous lesions rapidly improved with short course of oral corticosteroids (1 mg/kg/day), whereas kidney function and proteinuria remained unchanged despite bevacizumab discontinuation, supporting a superimposed gemcitabine-associated cTMA with tubular lipidosis in the setting of a drug-induced CryoGN, representing a unique and previously undescribed spectrum of renal toxicity.

### Clinical Follow-up

Following complete resolution of purpuric vasculitic lesions with corticosteroid therapy, rapid tapering and discontinuation were undertaken. In contrast, persistent proteinuria was considered a chronic renal sequela rather than a manifestation of active kidney disease, prompting the oncologic decision to resume bevacizumab therapy and optimize antiproteinuric therapy with angiotensin receptor blocker and an SGLT2 inhibitor, resulting in a subsequent marked reduction in proteinuria (0.5 g/24 h, serum creatinine 1.3 mg/dL).

## Discussion

Comprehensive integration of tissue findings with direct and indirect serologic markers is crucial in these complex settings. The coexistence of renal and cutaneous involvement, together with low C4 levels, elevated rheumatoid factor levels, and the pivotal ultrastructural evaluation of the polyclonal IgM intraluminal deposits in organized structure represented a strong diagnostic clue despite repeatedly negative circulating cryoglobulin assay, which may be limited by well-known preanalytical constraints (Table 1, Figs 2 and 3).^5^, ^6^, ^7^ An extensive etiologic workup is always required to exclude other secondary causes, including viral infections, autoimmune diseases, and lymphoproliferative disorders. Although seronegative cases has been described, nonhematologic, nonhepatitis-associated forms are exceedingly rare, often renal limited, and frequently underdiagnosed.^8^ Moreover, paraneoplastic cryoglobulinemia has been rarely reported in association with ovarian carcinoma, and the temporal relationship with chemotherapy exposure and the coexistence of gemcitabine-related TMA in our case favor a drug-induced etiology.^9^ Indeed, after a careful exclusion of alternative secondary causes, the present case was most plausibly due to a drug-induced cryoglobulin-like vasculitic syndrome, expanding the spectrum of kidney involvement associated with anticancer therapies. Indeed, there are no data describing CryoGN in patients treated with carboplatin, gemcitabine, and bevacizumab.^10^^,^^11^ The available clinical trials and safety evidence for these agents, including their use in combination regimens for ovarian, urothelial, and renal cancers, report renal adverse events, such as proteinuria, hypertension, and nephrotic syndrome, but do not document cases association with cryoglobulinemia or cryoglobulinemic vasculitis.^10^, ^11^, ^12^, ^13^, ^14^, ^15^, ^16^

**Figure 4:**
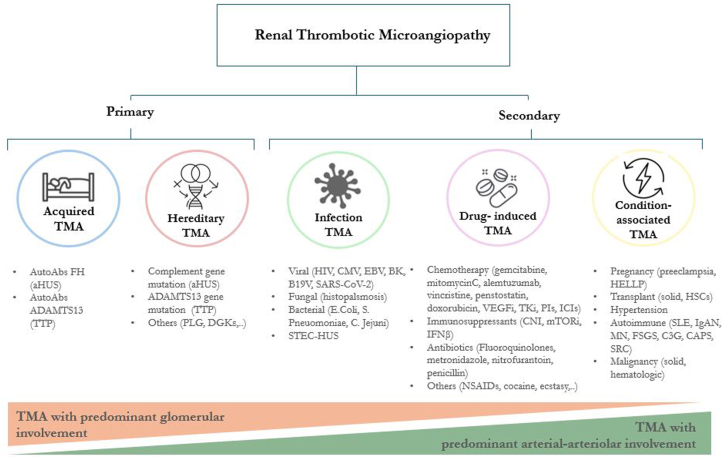
Renal thrombotic microangiopathy: a shared pathologic lesion. Abbreviations: TMA, thrombotic microangiopathy; aHUS, atypical hemolytic uremic syndrome; AutoAbs, autoantibodies; BK, B19V, parvovirus B19; C3G, C3 glomerulopathy; CAPS, catastrophic antiphospholipid syndrome; CMV, cytomegalovirus; CNI, calcineurin inhibitor; EBV, Epstein-Barr virus; FH, Factor H; FSGS, focal segmental glomerulosclerosis; HELLP, hemolysis, elevated liver enzymes and low platelets; HIV, human immunodeficiency virus; ICIs, immune checkpoint inhibitors; IFNβ, interferon β; IgAN, IgA nephropathy; MN, membranous nephropathy; mTORi, mammalian target of rapamycin inhibitors; NSAIDs, non-steroidal anti-inflammatory drugs; PIs, proteasome inhibitors; SLE, systemic lupus erythematosus; SRC, scleroderma renal crisis; STEC-HUS, Shiga toxin-producing *Escherichia coli* hemolytic uremic syndrome; TKi, tyrosine kinase inhibitors; TTP, thrombotic thrombocytopenic purpura; VEGFi, vascular endothelial growth factor inhibitors.

Renal thrombotic microangiopathy (TMA) encompasses multiple etiologies with overlapping morphologic features and variable clinical manifestations, including microangiopathic hemolytic anemia, thrombocytopenia, and renal dysfunction. Acute TMA shows fibrin-rich thrombi within capillaries and arterioles with endothelial swelling and depletion, whereas cTMA reflects sustained or delayed endothelial injury, characterized by a low-cellularity MPGN pattern with diffuse GBM duplication and mesangiolysis. The differential diagnosis of TMA is broad and relies on careful clinicopathologic correlation. Primary TMAs, such as thrombotic thromboytopenic purpura and hemolytic uremic syndrome, are predominantly glomerular, whereas secondary TMAs primarily affect arteries and arterioles. However, these patterns are not mutually exclusive and frequently overlap (Fig 4).^17^ In cancer therapy, drug-induced TMA comprises 2 forms. Type I, caused by cytotoxic agents such as gemcitabine, is dose dependent, delayed, and frequently irreversible because of cumulative endothelial damage. Type II, related to vascular endothelial growth factor inhibition, is not dose dependent and often reversible after drug discontinuation. Possible complement dysregulation have been proposed as contributing factors, raising the possibility of anticomplement therapy in refractory cases, although supporting evidence remains limited.^18^

In our case, kidney biopsy showed a delayed and complex nephrotoxic pattern of lesions with diffuse mesangiolysis, severe endothelial loss, and advanced MPGN remodeling with extensive GBM duplications, without active thrombotic microangiopathy. This is consistent with a chronic, dose-dependent TMA. This is supported by the insidious clinical course, characterized by a gradual decline in kidney function and progressive hypertension over approximately 6-8 months, consistent with the current literature.^19^ The delayed temporal relationship with gemcitabine exposure and the predominance of chronic lesions favor gemcitabine-induced type I TMA over bevacizumab-related injury. This interpretation is further reinforced by the absence of recurrent acute renal impairment or worsening proteinuria after bevacizumab resumption. The persistent proteinuria, attributed to chronic injury and unresponsive to steroid therapy, was subsequently managed successfully with antiproteinuric nephroprotective treatment. Moreover, a particularly intriguing and previously unreported aspect of renal toxicity was the detection of myelin bodies (MBs) on electron microscopy in this clinical context. MBs are lamellated, electron-dense lysosomal inclusions because of phospholipid accumulation, classically seen in inherited lysosomal storage disorders, such as Fabry disease, or in nongenetic settings, after exposure to cationic amphiphilic drugs (Table 2).^20^^,^^21^ By contrast, oncologic therapy related renal injury is not typically associated with tubular MB formation, and ultrastructural descriptions linked to antineoplastic agents are lacking.^3^ In the present case, genetic testing for lysosomal storage disorders was negative, and prior exposure to the administered anticancer agents has not been reported to be associated with lysosomal pathway alterations. Among nononcologic drugs (Box 1), amlodipine was the only long-term exposure potentially associated with phospholipidosis, but its chronic use with preserved kidney function makes it an unlikely cause. Furthermore, the identification of tubular MBs on electron microscopy in this clinical context is of particular significance and warrants careful interpretation. Indeed, in the kidney, iatrogenic MBs are most frequently described within podocytes, whereas involvement of tubular epithelial cells is relatively uncommon.^21^ Although acute tubular injury is a recognized complication of several chemotherapeutic agents, lamellated lysosomal inclusions have not been established as a characteristic ultrastructural feature of chemotherapy-associated nephrotpxocity.^22^ Thus, the tubular MB observed represents a previously unrecognized manifestation of tubular chemotherapy-related injury expanding the spectrum of onco-nephrology pathology and underscoring the value of electron microscopy in identifying atypical patterns of renal drug toxicity undetectable by conventional histology (Fig 2).

**Table 2:** Medications Associated with Myeloid Body Formation.

Drug Class	
Antimicrobial agents	Aminoglycosides, Azithromycin, Chloramphenicol, Ketoconazole, Pentamidine, Nitrofurantoin
Antimalarial/antiparasitic agents	Chloroquine, Hydroxychloroquine, Mefloquine, Quinacrine
Cardiovascular agents	Amlodipine, Amiodarone, Atropine, beta-blockers, Quinidine, Ranolazine
Psychotropic agents	Chlorpromazine, Clozapine, Haloperidol, Thioridazine, Paroxetine, Sertraline
Antihistamines	Diphenhydramine, Hydroxyzine, Chlorcyclizine, Loratadine
Gastrointestinal agents	Pantoprazole, Loperamide, Alverine
Immunomodulatory agents	Tamoxifen, Vinblastine, Methotrexate
Lipid-lowering agents	Statins, Fenofibrate
Neurologic agents	Gabapentin, Memantine, Bromocriptine, Ropinirole
Hormonal agents	Progesterone, Mifepristone
Others	Contrast media, Silicone, Warfarin, Retinol

This case highlights an unusual and complex renal pathology, characterized by chronic gemcitabine-induced TMA with a drug related CryoGN and associated renal lipidosis. As the first report of this injury spectrum in this setting, it emphasizes the crucial role of kidney biopsy and ultrastructural evaluation in elucidating multifactorial nephrotoxicity and optimizing personalized oncological care.
